# Predictors of safety margin for coracoid transfer: a cadaveric morphometric analysis

**DOI:** 10.1186/s13018-019-1212-z

**Published:** 2019-06-10

**Authors:** Terufumi Shibata, Teruaki Izaki, Satoshi Miyake, Nobunao Doi, Yasuhara Arashiro, Yozo Shibata, Yutaka Irie, Katsuro Tachibana, Takuaki Yamamoto

**Affiliations:** 1 Department of Orthopaedic Surgery, Fukuoka University Faculty of Medicine, 7-45-1 Nanakuma, Jonan-ku, Fukuoka, 814-0180 Japan; 2 Department of Orthopaedic Surgery, Fukuoka University Chikushi Hospital, 1-1-1 Zokumyoin, Chikushino, Fukuoka, 818-8502 Japan; 3 Department of Anatomy, Fukuoka University Faculty of Medicine, 7-45-1 Nanakuma, Jonan-ku, Fukuoka, 814-0180 Japan

**Keywords:** Latarjet, Coracoid, Anatomy, Coracoid transfer, Height, Coracoacromial ligament

## Abstract

**Background:**

The purpose of this study was to investigate the relationship between the bone length available for coracoid transfer without coracoclavicular ligament injury and the distance from the coracoid tip to the attachments of the coracoacromial ligament or pectoralis minor. We hypothesized that cadaver height and the soft tissue attachments on the coracoid process were predictive factors for sufficient bone length for coracoid transfer.

**Methods:**

This study included 28 shoulders from Japanese cadavers: 19 male and 9 female. The distance from the coracoid tip to the distal attachment of the coracoclavicular ligament and the anterior and posterior margins of the coracoacromial ligament or pectoralis minor on the coracoid process were measured.

**Results:**

The mean available length for coracoid transfer was 24.8 ± 3.4 mm. There was a significant difference in length between male and female subjects, being 26.0 ± 2.9 mm and 22.2 ± 3.0 mm, respectively (*p* = 0.004). High positive correlations were found between the length of the coracoid transfer and cadaver’s height (*r* = 0.48, *p* = 0.009) and the distance from the coracoid tip to the anterior coracoacromial ligament attachment (*r* = 0.63, *p* < 0.001). The receiver operating characteristic curve area under the curve for cadaver height was 0.72 while that for distance from coracoid tip to anterior coracoacromial ligament was 0.88 when predicted for a sufficient length for coracoid transfer > 25 mm.

**Conclusions:**

Our findings will aid surgeons in preoperative planning and performing of osteotomy of the coracoid safely by predicting the available length of coracoid bone graft.

## Background

The Latarjet procedure provides shoulder stability for the treatment of anteroinferior shoulder instability via its triple blocking effect, and its long-term outcomes are reportedly excellent [1]. However, when performing the open Latarjet procedure recommended by Walch, a coracoid bone graft length of more than 25 mm is necessary to enable the safe insertion of two 4.5-mm screws into the bone graft [2, 3]. In cadaveric studies, the available bone graft length for coracoid transfer without coracoclavicular ligament injury was reported as 28.5 mm [4] and 26.4 mm [5]. Lian et al. [6] reported a mean length of the coracoid bone graft of 23.9 mm for Mongolian male cadavers, and this length was shorter than previous studies [4, 5]. The freedom from coracoclavicular injury (safety margin) of the coracoid process osteotomy level was different for individuals, meaning the amount of bone available for the coracoid transfer was limited. The coracoclavicular ligament attachment sites on the coracoid process are in the deep layer of the surgical field [7–10], so confirming them under direct views is sometimes difficult. Although the maximal length of the coracoid bone should be harvested to decrease the risk of complications, the surgeon may shorten the harvested coracoid bone graft to avoid potentially injuring the coracoclavicular ligament attachment [3, 11]. For preoperative planning, Armitage et al. [12] reported that computed tomography enabled the measurement of the length of the coracoid process from the tip to the elbow, which was considered the bony landmark of the osteotomy [13]. However, this method did not consider the attachment sites of the coracoclavicular ligaments and may therefore lead to injury of these ligaments. Therefore, the prediction of the safety margin of the coracoid is necessary to evaluate whether sufficient coracoid length can be obtained to achieve safe coracoid osteotomy.

The purpose of this study was to investigate the relationship between the length of bone available to enable coracoid transfer without coracoclavicular ligament injury and the distance from the coracoid tip to the attachments of the coracoacromial ligament or pectoralis minor. We hypothesized that a cadaver’s height and the soft tissue attachments on the coracoid process were predictive factors for sufficient bone length for coracoid transfer.

## Methods

**Fig. 1:**
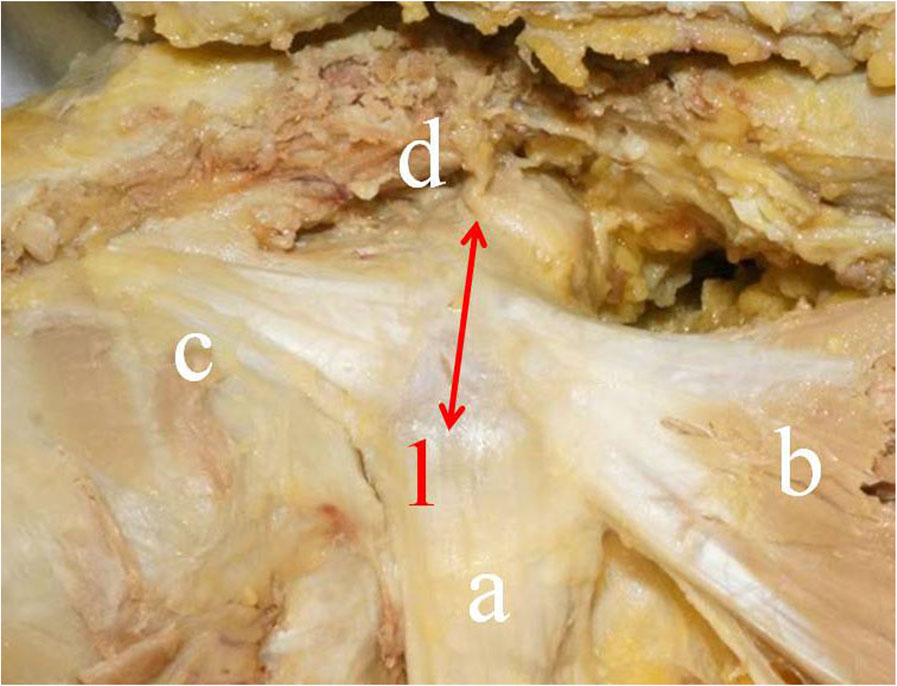
Anatomic soft tissue attachments on the coracoid process of the right shoulder. (**a**) Conjoint tendon. (**b**) Pectoralis minor. (**c**) Coracoacromial ligament. (**d**) Trapezoid ligament. 1 indicates coracoid tip to distal attachment of trapezoid ligament

Twenty-eight shoulders in Japanese cadavers (19 male and 9 female) provided to the Department of Anatomy of our institution were investigated. The study was approved by our Institutional Review Board. The mean (± standard deviation) age at the time of death was 75.6 ± 12.3 years, and the mean donor height when alive was 163.1 ± 7.0 cm for men and 151.0 ± 5.9 cm for women. Cadavers were preserved in formalin-based dilution, and none had had previous surgery or fracture around the shoulder. The skin, subcutaneous tissues, deltoid, and pectoralis major muscle were removed from the shoulder. This exposed the coracoid process, pectoralis minor muscle, conjoint tendon, coracoacromial ligament, and coracoclavicular ligament (Fig. 1). Each ligament and tendon attachment area was clearly identified and precisely prepared.

**Fig. 2:**
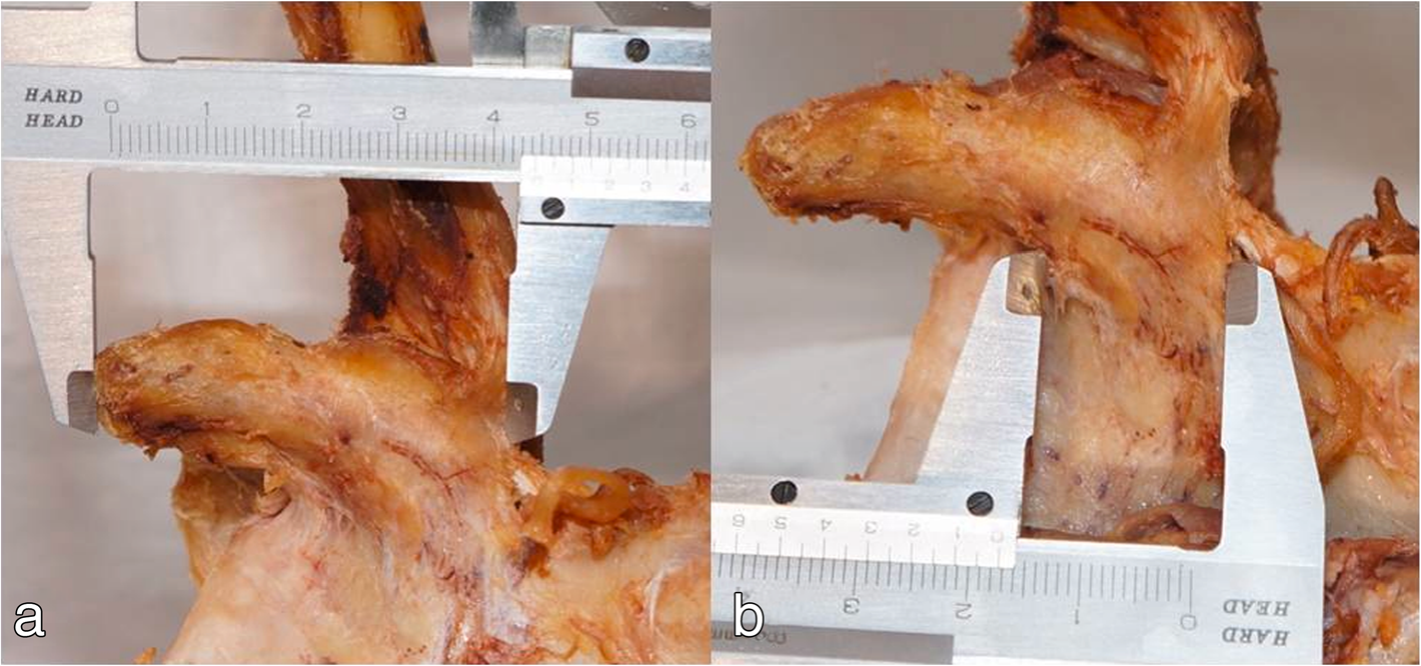
Anteromedial view of the right shoulder showing the measurement of the total coracoid length (**a**) and of the length of the coracoid base (**b**)

The distances from the coracoid tip to the distal attachment of the trapezoid ligament and to the anterior and posterior margin of the pectoralis minor or coracoacromial ligament were measured. The bony landmarks of the coracoid process were also measured, including the total coracoid length and the coracoid base length (Fig. 2a, b). Assuming an osteotomy level at the coracoid elbow, the mean coracoid length available for transfer, such as the distance from the coracoid tip to the elbow, was calculated [(total coracoid length) − (coracoid base length)]. All geometric measurements were recorded with calipers accurate to 0.1 mm, and these measurements were analyzed by gender. We also assessed the potential correlation between these measurements and the cadavers’ heights. The relationship between the soft tissue attachments on the coracoid process and the length of the coracoid transfer were also evaluated.

### Statistical analysis

Statistical analyses were performed using IBM SPSS, version 21 (IBM, Armonk, NY). Data were expressed as means ± standard deviations. For all independent variables, Shapiro-Wilk’s test of normality was used. A paired *t* test for normally distributed data or the Mann-Whitney *U* test for non-normally distributed data were used to define differences. To evaluate the strength of the relationship between corresponding variables, Pearson’s correlation was performed for normally distributed variables and Spearman rank correlation for variables without a normal distribution. *p* < 0.05 was considered statistically significant. To determine a formula that was predictive of sufficient length of the coracoid transfer > 25 mm, receiver operating characteristic (ROC) curves were performed with the use of 95% confidence intervals (CIs) for the variables of cadaver height and the distances from the coracoid tip to soft tissue attachments.

## Results

Geometric data of the coracoid and their relation to the coracoid soft tissue attachments are summarized in Table 1. Significant differences between males and females were found for the distances from the coracoid tip to the distal trapezoid ligament and elbow and for total coracoid length. The length from the coracoid tip to the posterior pectoralis minor and the coracoid base length tended to be longer in males, but no statistical difference was found for either. Other measurements for the soft tissue attachments to the coracoid bony landmarks were also comparable between males and females. When considering the mean length of the coracoid available for transfer, the mean distance from the coracoid tip to the distal trapezoid ligament was shorter than that from the coracoid tip to the elbow (*p* = 0.011). There was an available coracoid length of < 25 mm in 4 shoulders (21.0%) in males and 6 shoulders (66.7%) in females. However, if the osteotomy was performed at the elbow of the coracoid, a coracoidal bone length of < 25 mm was present in 1 shoulder (5.3%) in males and 6 shoulders (66.7%) in females.

**Table 1:** Geometric data of the coracoid and their relation to the coracoid soft tissue attachments

Characteristic, mean ± SD (range)	Total (*n* = 28)	Male (*n* = 19)	Female (*n* = 9)	*p* value
Total coracoid length (mm)	44.3 ± 4.1 (33.8–50.2)	46.1 ± 3.5 (33.8–50.2)	40.7 ± 2.8 (35.8–44.4)	< 0.001
Coracoid base length (mm)	17.3 ± 1.8 (11.9–21.1)	17.8 ± 1.5 (15.2–21.1)	16.4 ± 2.0 (11.9–18.3)	0.05
Coracoid tip to elbow (mm)	27.0 ± 3.8 (15.7–31.2)	28.3 ± 3.4 (15.7–31.2)	24.3 ± 3.2 (18.7–30.5)	0.001
Coracoid tip to distal trapezoid ligament (mm)	24.8 ± 3.4 (17.7–29.8)	26.0 ± 2.9 (17.7–29.8)	22.2 ± 3.0 (18.5–27.0)	0.004
Coracoid tip to anterior pectoralis minor (mm)	6.6 ± 2.8 (1.4–11.8)	6.8 ± 2.7 (1.4–11.8)	6.1 ± 2.9 (2.5–10.8)	0.52
Coracoid tip to posterior pectoralis minor (mm)	19.2 ± 4.1 (12.3–29.4)	20.1 ± 4.3 (14.4–29.4)	17.3 ± 3.3 (12.3–22.7)	0.10
Coracoid tip to anterior coracoacromial ligament (mm)	6.8 ± 2.0 (3.0–10.8)	7.2 ± 2.0 (3.3–10.8)	6.1 ± 1.9 (3.0–9.0)	0.20
Coracoid tip to posterior coracoacromial ligament (mm)	20.7 ± 6.3 (13.5–35.1)	19.8 ± 6.3 (13.5–35.1)	22.6 ± 6.1 (14.6–32.0)	0.24

*SD* standard deviation

The correlations of cadaver heights and coracoid soft tissue attachments with various coracoid distances are shown in Table 2. Distances from the coracoid tip to the distal trapezoid ligament and to the elbow, and total coracoid length, were significantly correlated with cadaver heights. A positive statistically significant relationship was found between the distance from the coracoid tip to the distal trapezoid ligament and the distance from the coracoid tip to the anterior coracoacromial ligament (Table 3).

**Table 2:** Correlation of cadaver height and the soft tissue attachment on the coracoid process

Cadaver height vs	*r*	*p* value
Total coracoid length	0.59	0.001
Coracoid base length	0.39	0.04
Coracoid tip to elbow	0.51	0.005
Coracoid tip to distal trapezoid ligament	0.48	0.009
Coracoid tip to anterior pectoralis minor	0.002	0.99
Coracoid tip to posterior pectoralis minor	0.23	0.24
Coracoid tip to anterior coracoacromial ligament	0.16	0.40
Coracoid tip to posterior coracoacromial ligament	-0.14	0.47

**Table 3:** Correlation of safety margin with the soft tissue attachments on the coracoid process

Coracoid tip to distal trapezoid ligament vs	*r*	*p* value
Coracoid tip to anterior pectoralis minor	0.058	0.77
Coracoid tip to posterior pectoralis minor	0.26	0.19
Coracoid tip to anterior coracoacromial ligament	0.63	< 0.001
Coracoid tip to posterior coracoacromial ligament	0.062	0.75

**Fig. 3:**
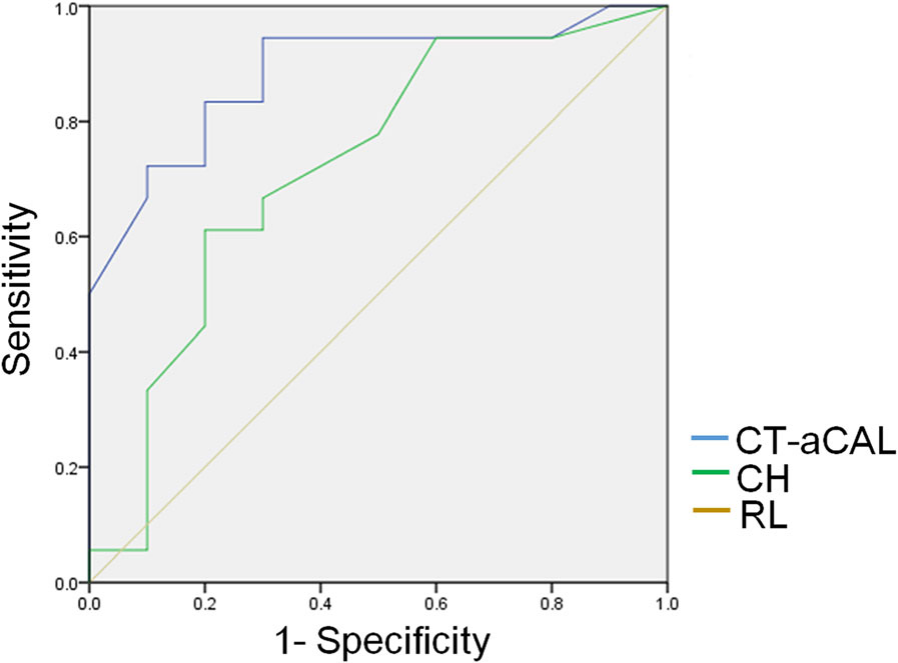
Receiver operating characteristic (ROC) curves for the cadaver’s height (CH) and distance from the coracoid tip to the anterior margin of the coracoacromial ligament (CT-aCAL). CT-aCAL had the highest calculated area under the curve. RL, reference line

The ROC curve in Fig. 3 shows the accuracy of the various factors for predicting sufficient bone length for coracoid transfer. Among soft tissue attachments on the coracoid, the strong association of the distance from the coracoid tip to anterior coracoacromial ligament with the sufficient length of the coracoid bone graft was reflected by the area under the curve (AUC) of 0.88 (95% CI, 0.76–1.0). The AUC of the ROC curve for the cadaver’s height for predicting sufficient length of the bone graft was 0.72 (95% CI 0.51–0.93). A distance from the coracoid tip to the anterior coracoacromial ligament of > 6.6 mm yielded a sensitivity of 83.3% and a specificity of 80.0% for sufficient length for coracoid transfer, while a cadaver height > 159.5 cm yielded a sensitivity of 61.1% and a specificity of 80.0% for sufficient length for coracoid transfer.

## Discussion

In the present study, we hypothesized that a cadaver’s height and the soft tissue attachments on the coracoid process were predictive factors for a sufficient bone graft length for coracoid transfer. This hypothesis was confirmed by the finding that cadaver height and the distance from the coracoid tip to the anterior coracoacromial ligament were associated with the length of the available coracoid bone graft. Furthermore, these two factors were moderately accurate measures [14] for predicting a sufficient length for the coracoid transfer of > 25 mm in the ROC curve analysis.

The safety margin for the coracoid osteotomy in our study was similar to the data derived from the male Mongolian donors [6], and these results of bone graft length were shorter than in previous studies [4, 5]. This difference in the safety margin was probably due to the differences in race and cadaver heights. Our study found high positive correlations between the safety margin and cadaver height. Because males were generally taller than females in our study, the safety margin for the coracoid osteotomy was proportionally longer in males. Asian people are relatively shorter than people in Western countries [15, 16], so the length of the bone available for coracoid transfer tends to be shorter. For the purpose of predicting the available coracoidal bone graft, measuring the distance from the coracoid tip to the elbow can be facilitated using preoperative computed tomography [12]. However, this method could overestimate the length of the coracoid transfer: the mean distance from the coracoid tip to the distal trapezoid ligament was shorter than the distance from the coracoid tip to the elbow in our study.

Terra et al. reported that the safety margin of the coracoid was correlated with the distance from the coracoid tip to the posterior margin of the pectoralis minor tendon [5]. However, the correlation of these two factors was not confirmed in our study. The pectoralis minor frequently originates from the second to fifth ribs and attaches to the anteromedial aspect of the coracoid process, but the mutation rate of the footprint of the pectoralis minor is reportedly 15 to 23.3% [6, 17]. Therefore, the possibility of pectoralis minor malformation should be considered when predicting the graft length available for the coracoid transfer. The present study found the safety margin of the coracoid was significantly correlated with the distance from the coracoid tip to the anterior margin of the coracoacromial ligament, which was easily identifiable intraoperatively. Various anatomic variants of the coracoacromial ligament were confirmed previously [18]. The attachment of the anterior margin of the coracoacromial ligament was constant regardless of the anatomic variants, but the attachment of the posterior margin of the coracoacromial ligament depended on variants [19]. For example, Y-shaped and V-shaped ligaments had more than one insertion point into the coracoid, and the most medial branch of the coracoacromial ligament formed the attachment of the posterior margin. For that reason, no significant correlation was observed between the safety margin of the coracoid and the distance from the coracoid tip to the posterior margin of the coracoacromial ligament.

This study demonstrated that a cadaver’s height and the distance from the coracoid tip to the anterior margin of the coracoacromial ligament were valuable measures for predicting a sufficient length for coracoid transfer of > 25 mm. A height > 159.5 cm or a distance from the coracoid tip to the anterior margin of the coracoacromial ligament > 6.6 mm was associated with sufficient length of the coracoid bone graft. A patient’s height can be checked during preoperative planning, and the distance from the coracoid tip to the anterior margin of the coracoacromial ligament can be easily measured intraoperatively. The coracoid elbow can be visualized intraoperatively, but the trapezoid ligament may not be completely visible; thus, attempting to preserve the attachment of this ligament can potentially limit the site of the osteotomy [3]. When performing the open Latarjet procedure, the harvested coracoid bone graft should be as large as possible to reduce complications such as graft nonunion and fracture [11]. The two predictive variables identified in our study (i.e., the patient height and the distance from the coracoid tip to the anterior margin of the coracoacromial ligament) may be used to predict the possibility of obtaining a sufficient coracoid bone graft length of > 25 mm. Furthermore, these two variables could be used to reduce the risk of limiting the site of the osteotomy. If these two predictors indicate that there will be an insufficient amount of coracoid bone available for the graft, the surgeon should change the operative method to the use of a 3.5-mm or 4.0-mm screw [20, 21], the Bristow procedure [22], or the use of structural bone graft options such as autologous iliac crest bone grafting [23].

There are several limitations to this study. First, our sample was small because we were permitted to use the shoulder from only one side of each cadaver because the cadavers were required for further medical education at our institution. Small sample size may have prevented detection of other relationships, such as that between the safety margin of the coracoid osteotomy and the distance from the coracoid tip to the posterior margin of the pectoralis minor. Second, our study was based on the operative technique of the open Latarjet procedure using two screws. The open Latarjet procedure using one screw and a washer also reportedly provides satisfactory clinical outcomes [24], and we consider this technique useful when a coracoid bone graft of > 25 mm cannot be obtained. However, single-screw fixation may not provide sufficient stability against rotation, which leads to graft nonunion or migration [25]. Therefore, further studies are needed to determine which fixation method is appropriate. Third, it was difficult to measure the distance from the coracoid tip to the elbow because of the anatomical variations in shape and morphology of these structures [26]. We substituted the distance by calculating [(total coracoid length) − (coracoid base length)], which may have introduced measurement error. Finally, most cadavers in our study were older, and younger cadavers are generally more suitable for morphologic research because anteroinferior shoulder instability is a common disease in younger athletes.

## Conclusion

Our findings will aid surgeons in preoperative planning and in performing osteotomy of the coracoid safely by enabling prediction of the length of coracoid bone graft available.

## Data Availability

The datasets used during the current study are available from the corresponding author on reasonable request.

## Abbreviations

AUC: Area under the curve
CI: Confidence interval
ROC: Receiver operating characteristic
